# Supplementary material for: MYOC Promotes the Differentiation of C2C12 Cells by Regulation of the TGF-β Signaling Pathways via CAV1
Source: Biology (Basel). 2021 Jul 20;10(7):686. doi: 10.3390/biology10070686 (PMC8301362; doi:10.3390/biology10070686)
Supplement: Supplementary file 1 [file biology-10-00686-s001.zip › Supplementary Materials S2.pdf]

Supplementary Materials S2：

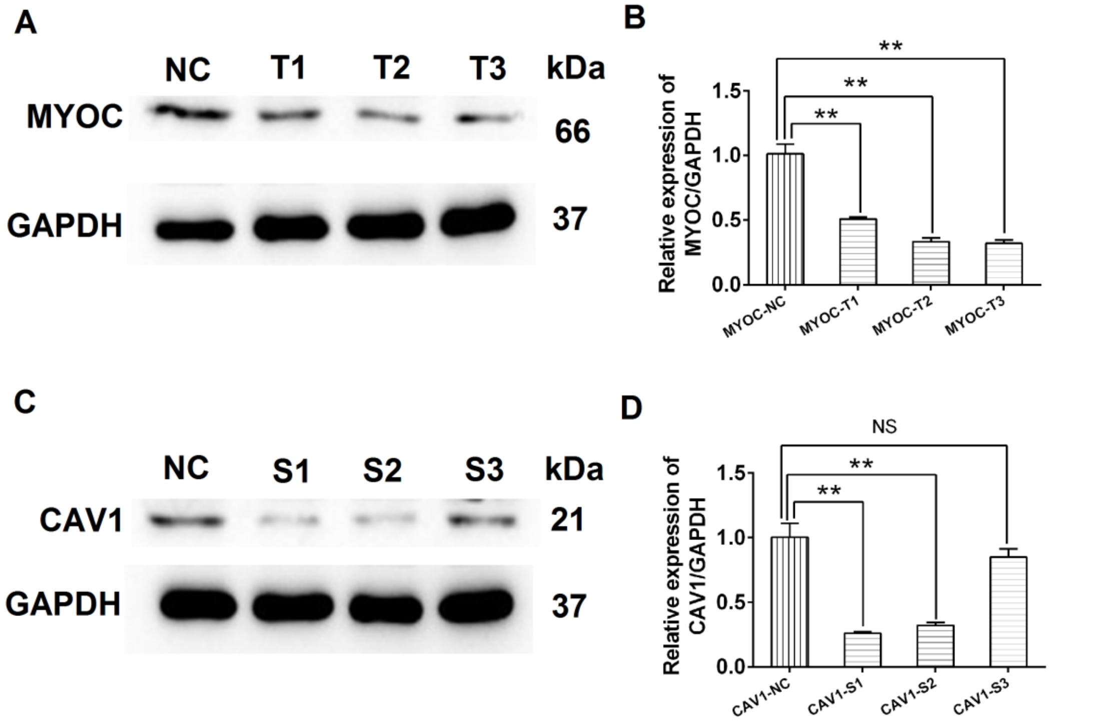

Supplementary Materials S2. Inhibits screening of fragments

Ralated to FIGURE 2 and FIGURE 4.

A. MYOC inhibited siRNA sequence screening. B. Grayscale scan of MYOC in Figure A. C. CAV1 inhibited siRNA sequence screening. D. Grayscale scan of CAV1 in Figure C.
