# Supplementary material for: MYOC Promotes the Differentiation of C2C12 Cells by Regulation of the TGF-β Signaling Pathways via CAV1
Source: Biology (Basel). 2021 Jul 20;10(7):686. doi: 10.3390/biology10070686 (PMC8301362; doi:10.3390/biology10070686)
Supplement: Supplementary file 1 [file biology-10-00686-s001.zip › Supplementary Materials S3.pdf]

Supplementary Materials S3：

Part of MYOC's immunoprecipitation mass spectrometry sequencing results

ProteinID	ProteinName
O54724	Caveolae-associated protein 1
Q60634	Flotillin-2
Q60875	Rho guanine nucleotide exchange factor 2
Q8CD09	LIM zinc-binding domain-containing protein
Q8CI51	PDZ and LIM domain protein 5
Q8BTS0	DEAD (Asp-Glu-Ala-Asp) box polypeptide 5
Q78PY7	Staphylococcal nuclease domain-containing protein 1
O08539	Myc box-dependent-interacting protein 1
B2RR82	Intersectin-2
A2AMM0	Caveolae-associated protein 4
P11087	Collagen alpha-1(I) chain
P53690	Matrix metalloproteinase-14
Q63918	Caveolae-associated protein 2
Q71LX8	Heat shock protein 84b
