# Supplementary material for: MYOC Promotes the Differentiation of C2C12 Cells by Regulation of the TGF-β Signaling Pathways via CAV1
Source: Biology (Basel). 2021 Jul 20;10(7):686. doi: 10.3390/biology10070686 (PMC8301362; doi:10.3390/biology10070686)
Supplement: Supplementary file 1 [file biology-10-00686-s001.zip › Supplementary Materials S1.pdf]

Supplementary Materials S1:

Manuscript title: **MYOC promotes the differentiation of C2C12 cells by regulation of the TGF-β signaling pathways via CAV1**

Information note: We used polyclonal antibodies in our experiment. So you can see several blots in some WB graphs, you can also see only one blot in some WB graphs because we cropped the NC membranes in WB process with the needs of experiments.

Western blotting raw data

Figure 1A

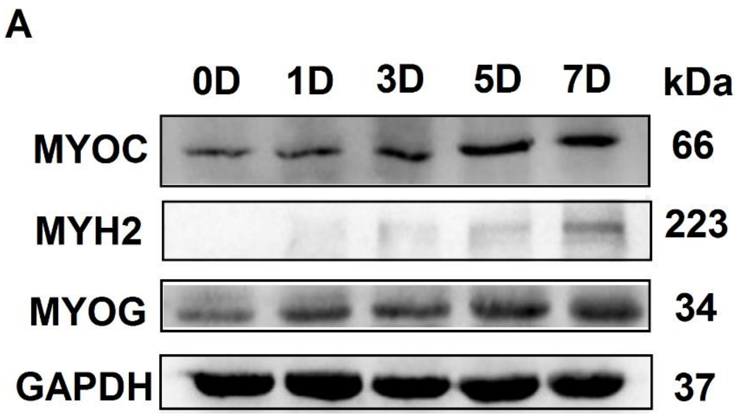

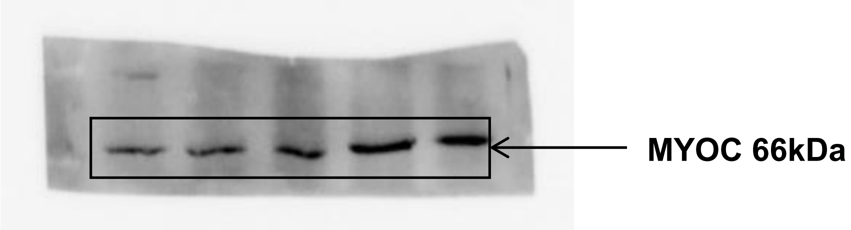

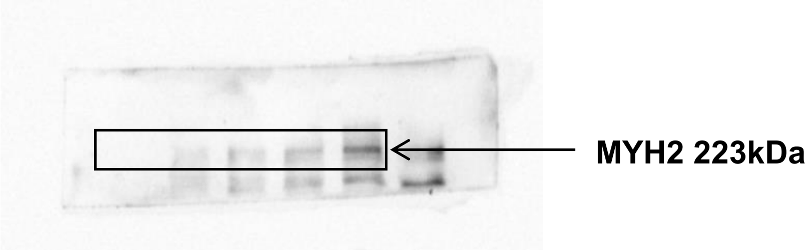

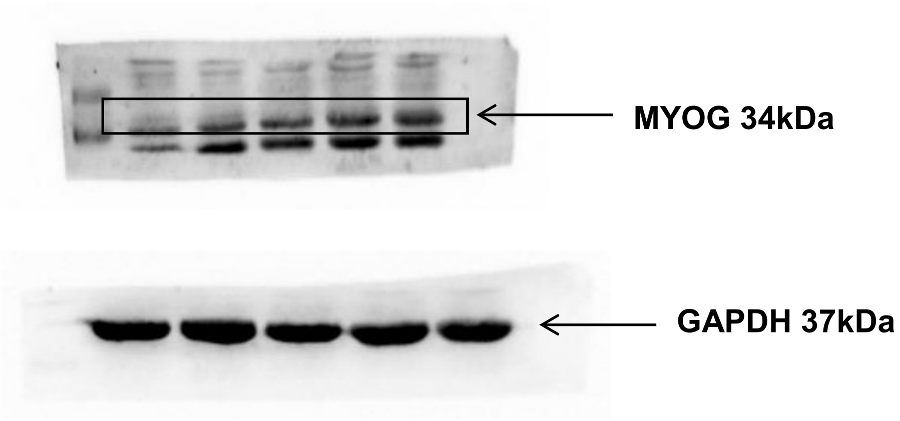

Western blotting raw data of Figure 1A

Figure 2A

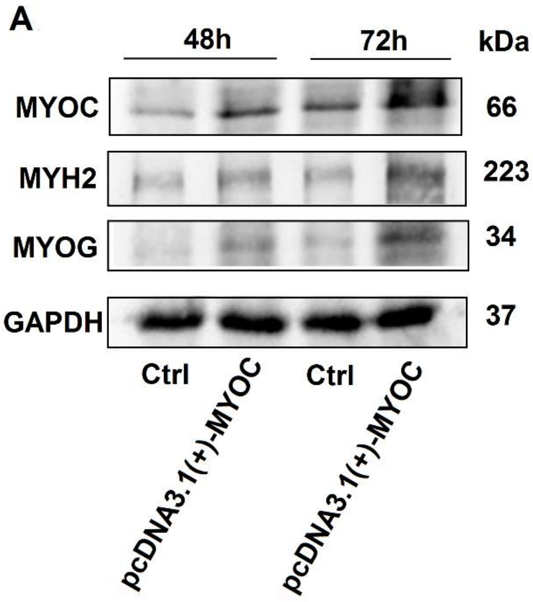

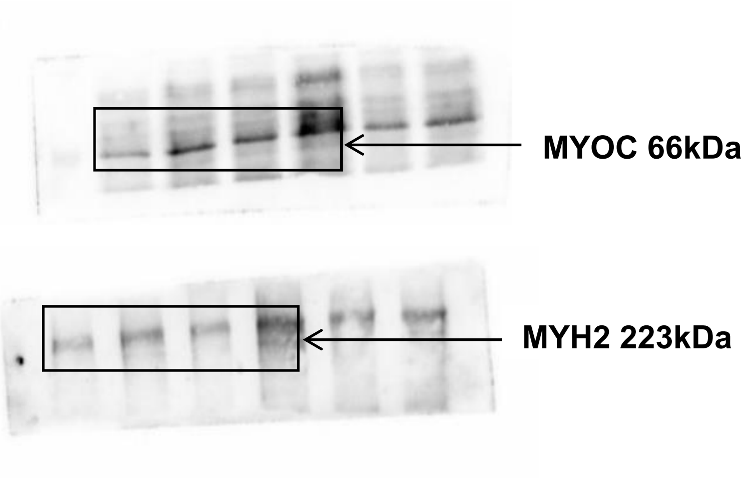

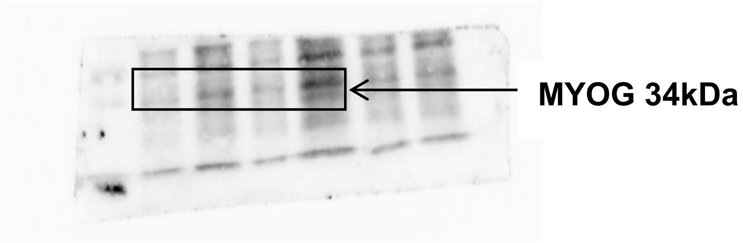

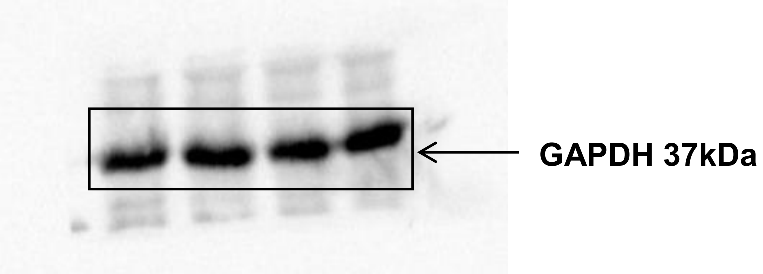

Western blotting raw data of Figure 2A

Figure 2G

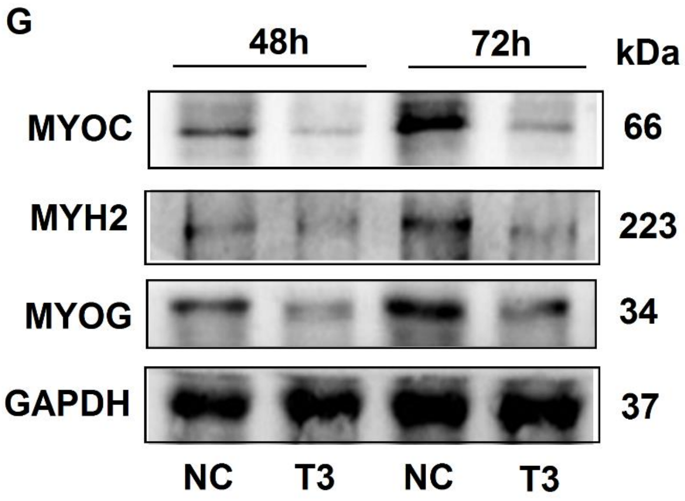

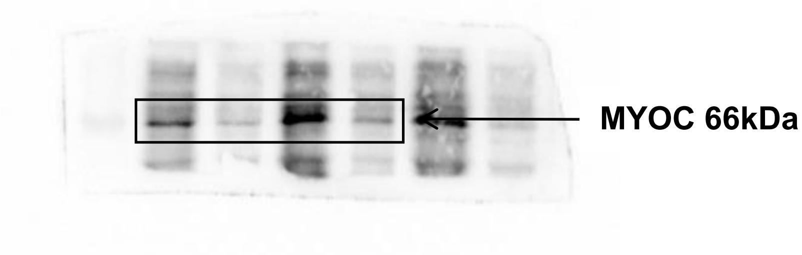

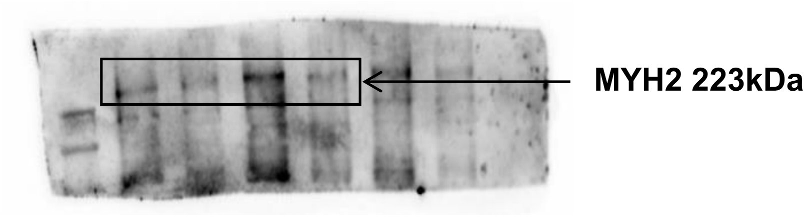

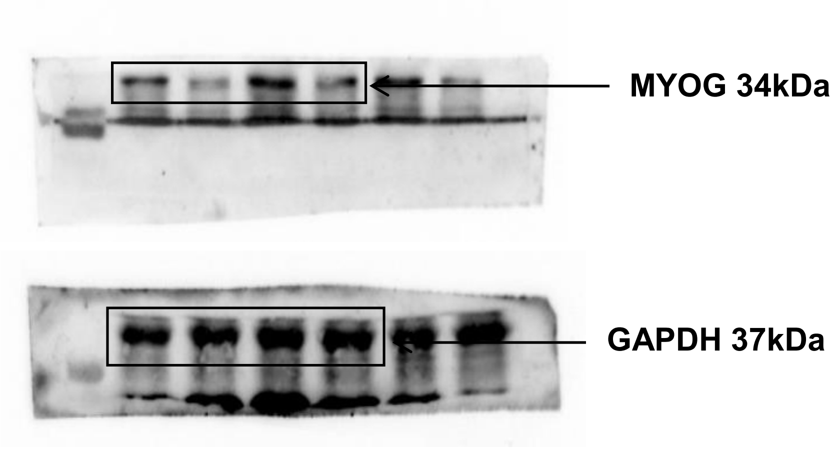

Western blotting raw data of Figure 2G

Figure 4A

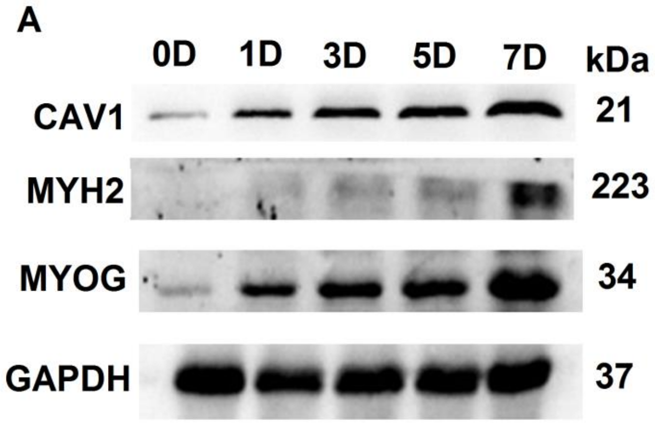

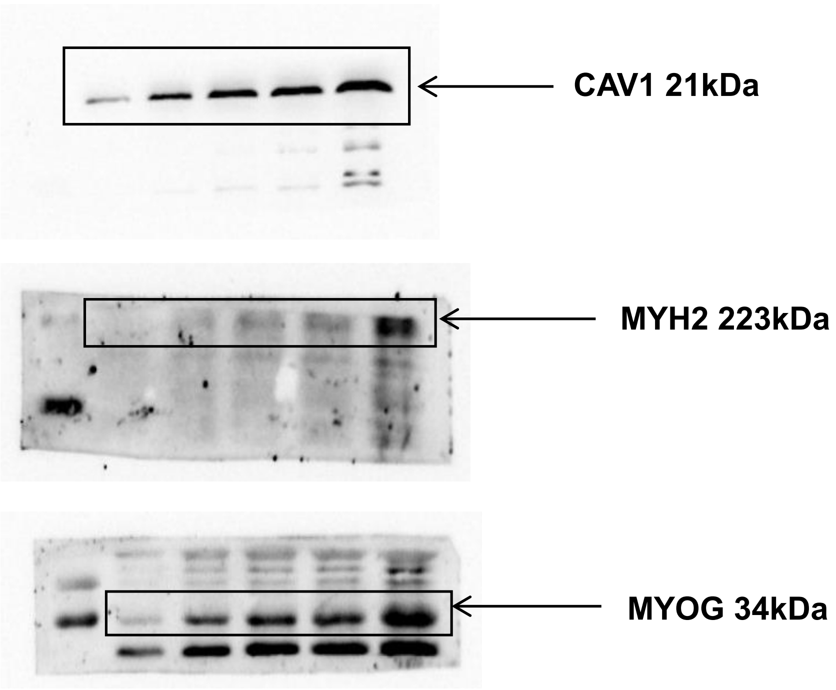

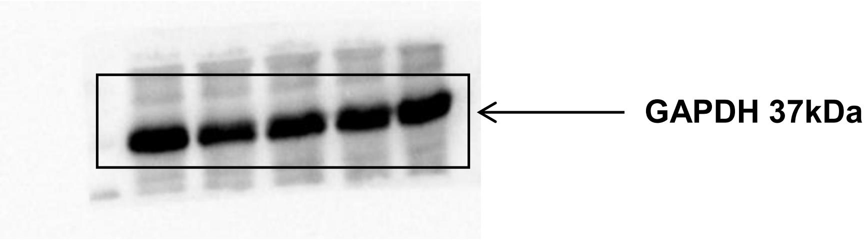

Western blotting raw data of Figure 4A

Figure 4G

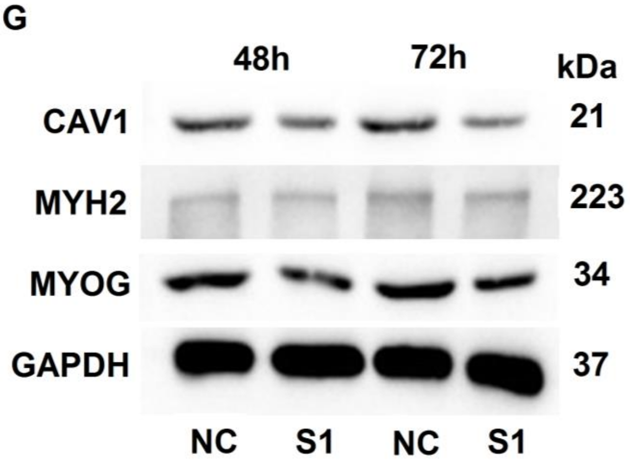

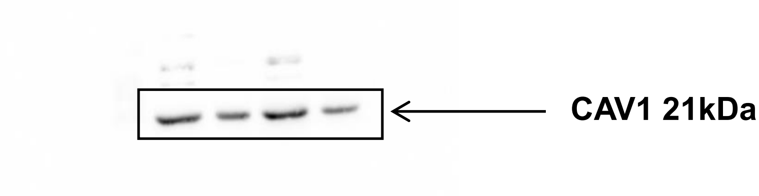

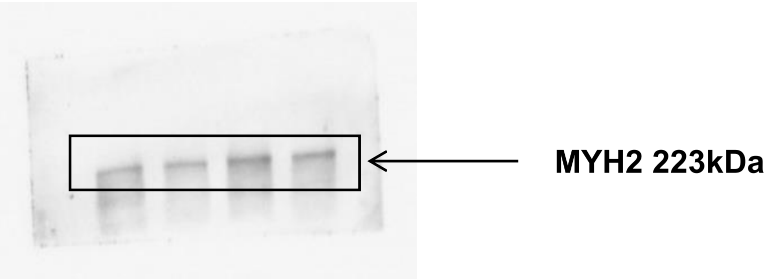

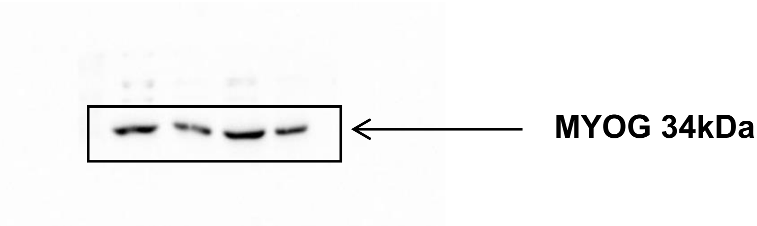

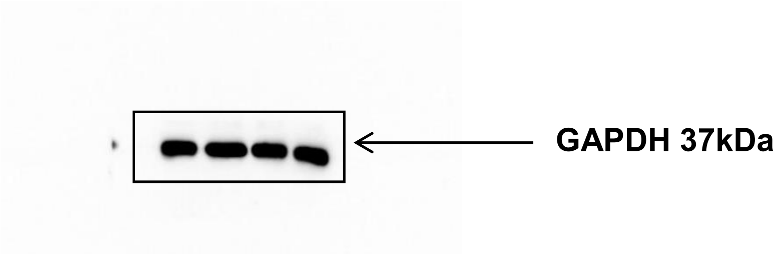

Western blotting raw data of Figure 4G

Figure 4M

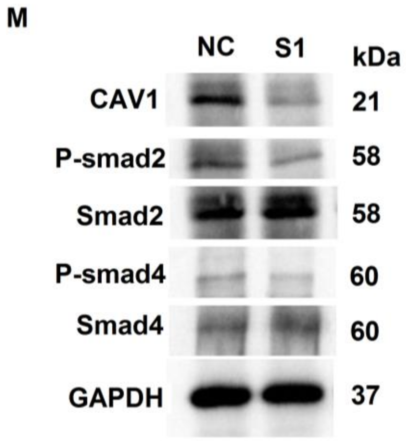

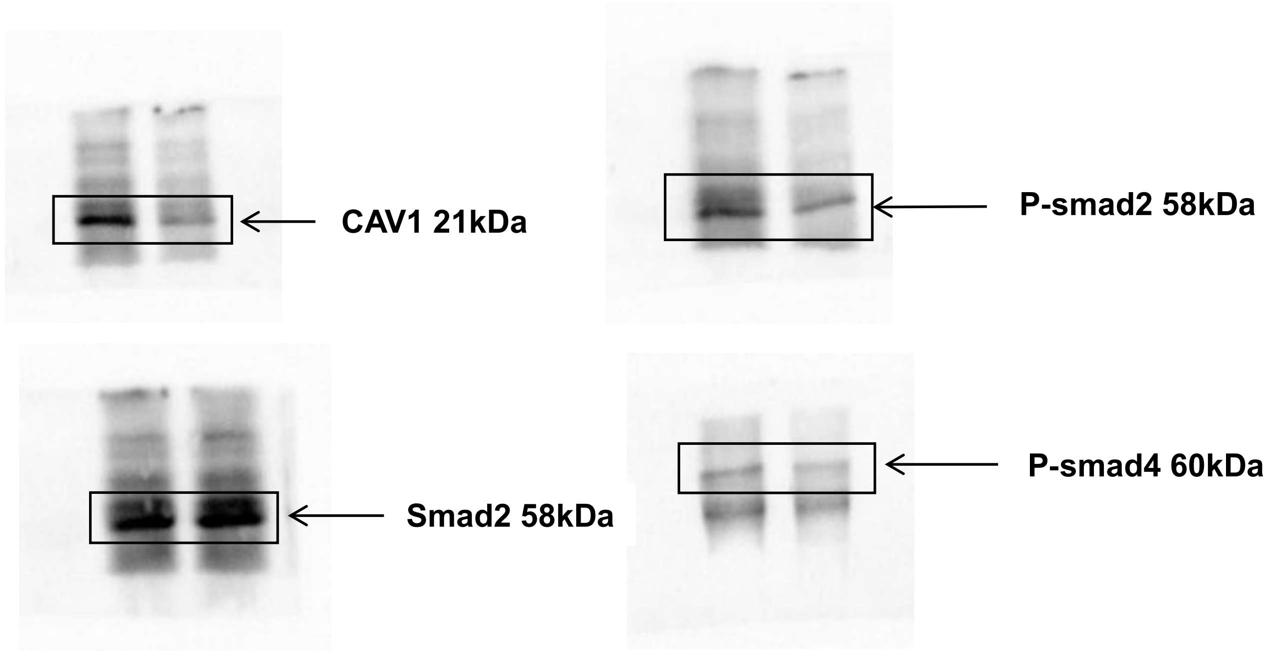

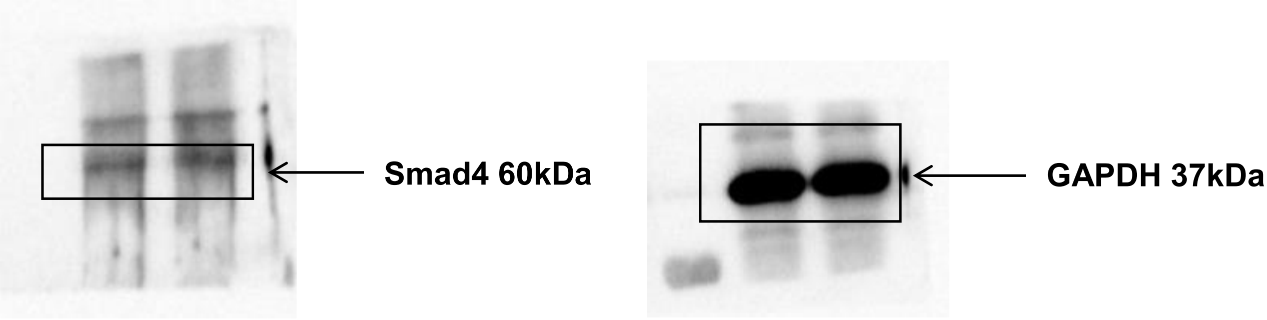

Western blotting raw data of Figure 4M

Figure 5A

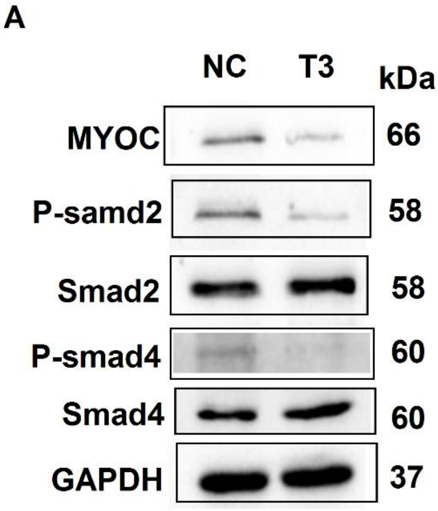

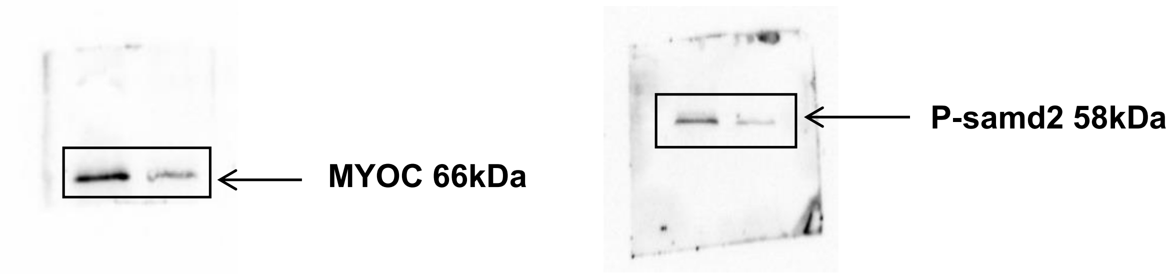

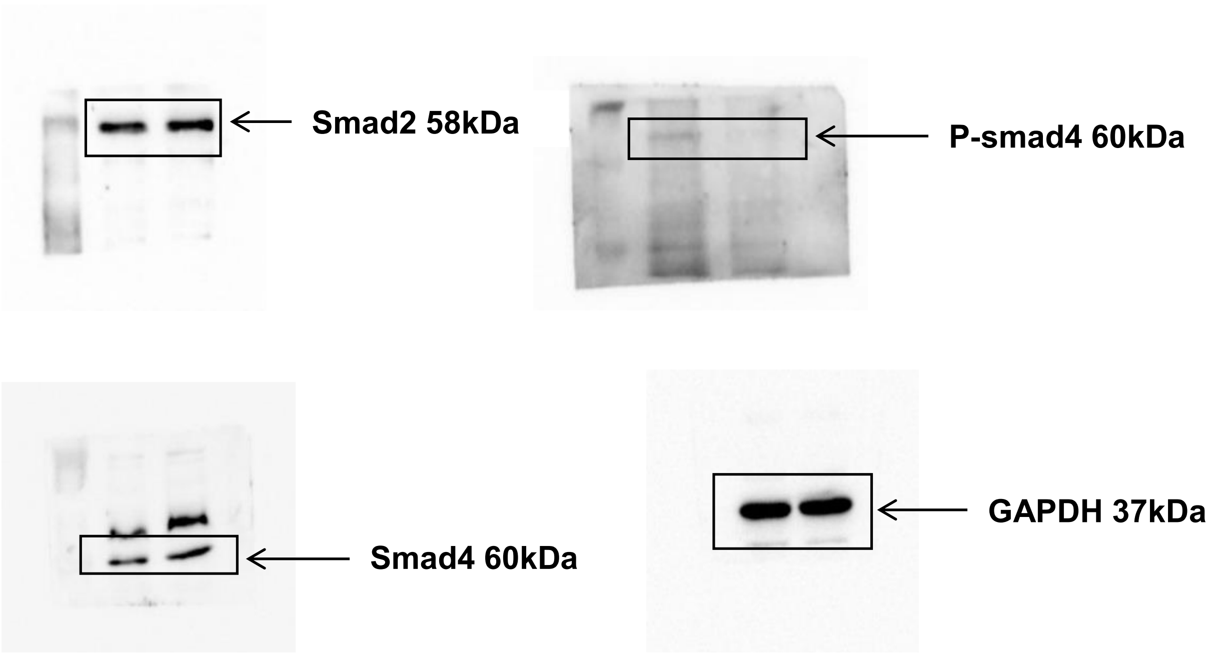

Western blotting raw data of Figure 5A

Figure 5E

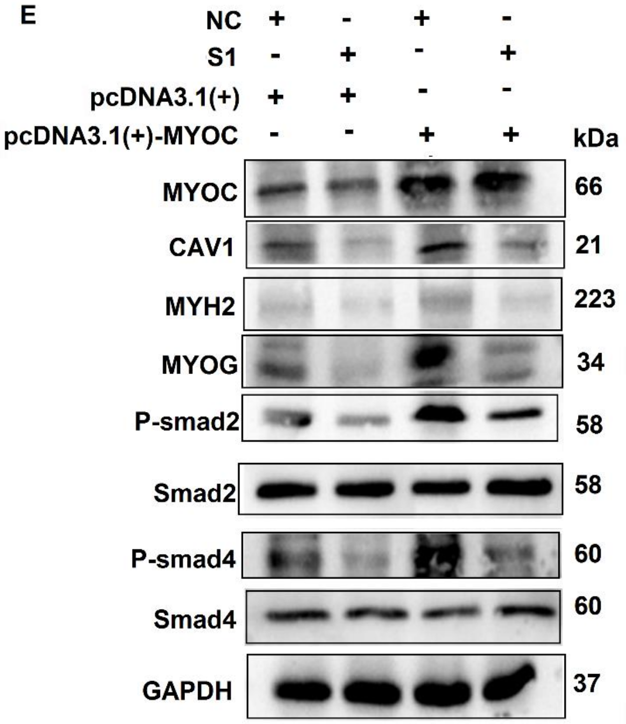

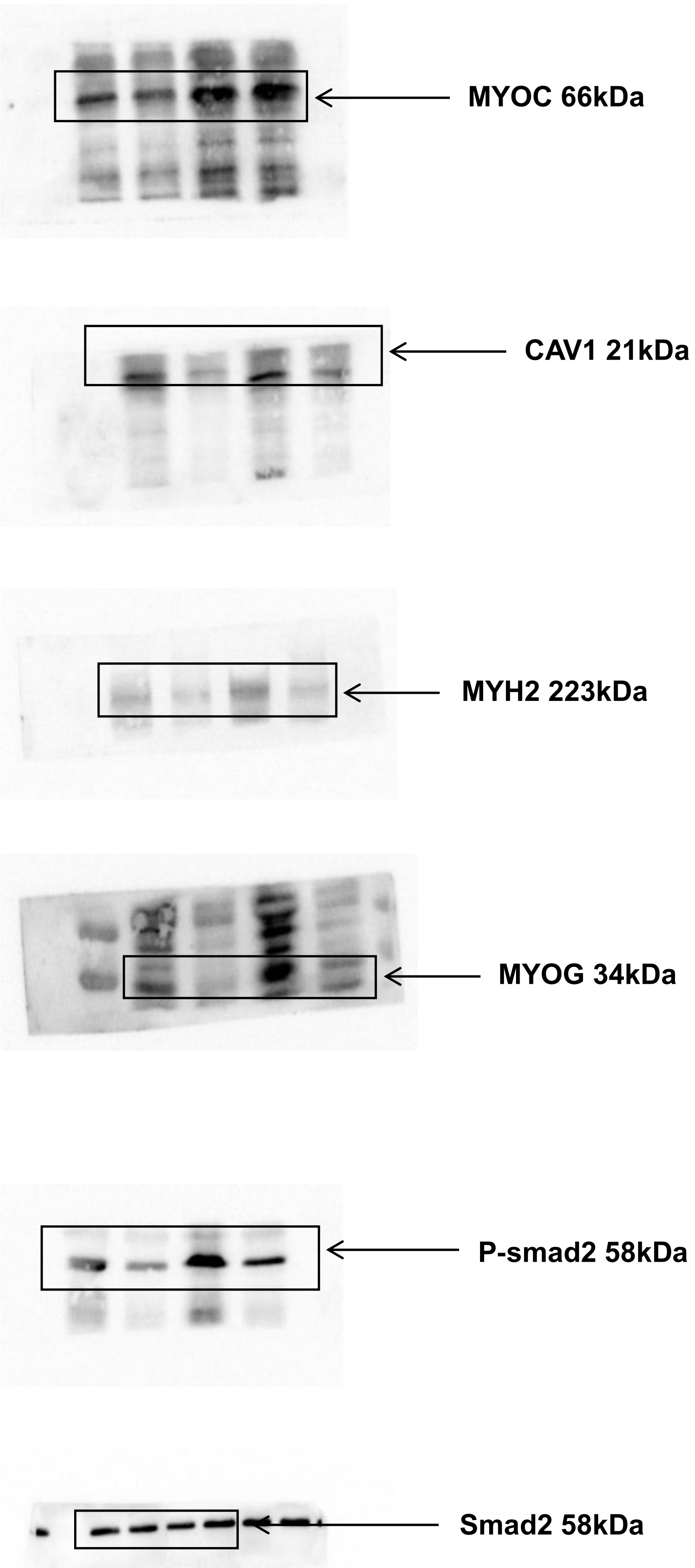
MYOC 66kDa
CAV1 21kDa
MYH2 223kDa
MYOG 34kDa
P-smad2 58kDa
Smad2 58kDa

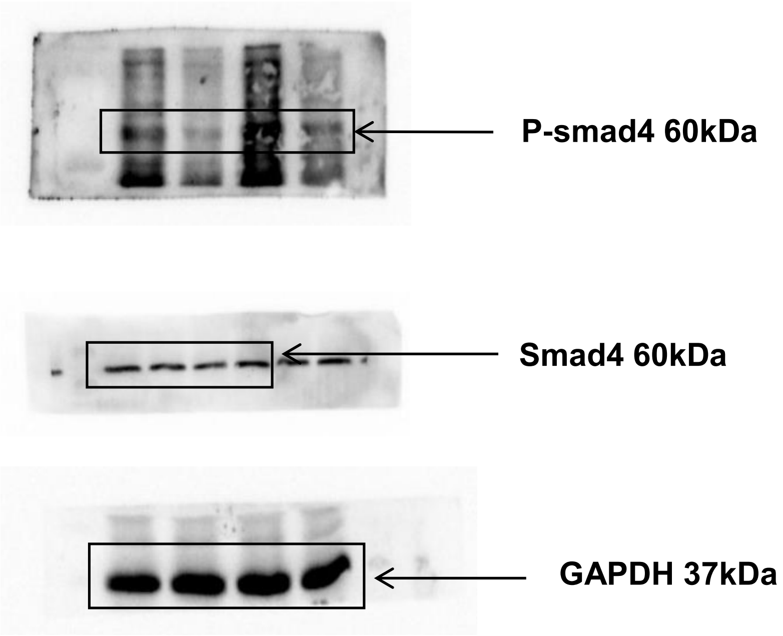

Western blotting raw data of Figure 5E
